# Supplementary material for: Are carbon emissions trading and green financial instruments synergistic? -Comprehensive quantitative research based on content analysis
Source: PLoS One. 2024 Mar 7;19(3):e0298601. doi: 10.1371/journal.pone.0298601 (PMC10919601; doi:10.1371/journal.pone.0298601)
Supplement: S1 Data — (ZIP) [file pone.0298601.s001.zip › Data/地级市Z_F工作报告.zip!/╝¬┴╓/╝¬┴╓╩╨/└┤╫╘╣1⁄2╓┌║┼í╛┬φ┐╦╩2╛▌═°í┐_2011.docx]

各位代表：

　　现在，我代表市政府向大会报告工作，请予审议，并请政协委员提出意见。

　　一、过去五年及2010年工作回顾

　　过去五年是我市发展历程上极不平凡的五年。在省委省政府和市委的正确领导下，在市人大、市政协的大力支持下，我们紧紧抓住加快老工业基地振兴的历史机遇，深入落实科学发展观，突出经济发展方式转变，坚持“四个坚定不移”，努力应对国际金融危机冲击等诸多严峻挑战，通过全力组织实施经济总量翻番、工业强市建设等重大战略，经济社会发展实现了历史性跨越。经济发展迈上新台阶，综合经济实力显著增强；产业结构不断优化，经济发展方式转变积极推进；各项改革深入推动，开发开放格局加速形成；城乡承载功能全面提升，城镇化发展进程有效加快；人民生活明显改善，社会建设和生态文明建设取得重大成果。地区生产总值年均增长24%，相当于2005年的3倍；固定资产投资年均增长35.4%；全口径财政收入年均增长23.1%，地方级财政收入年均增长25.4%；三次产业比重调整到9.8:50.6:39.6；城市居民人均可支配收入、农村居民人均纯收入年均增长13.9%和12.8%。“十一五”规划任务的胜利完成，为“十二五”时期更好更快发展奠定了良好基础。

　　去年，我市不仅面对后金融危机时代严峻复杂的外部环境，而且遭受了百年不遇的特大洪涝灾害。在各种困难尤为集中的情况下，我们牢牢把握转变经济发展方式这条主线，一手抓经济社会发展，一手抓灾后恢复重建，实现了经济社会的持续较快发展，夺取了抗洪抢险救灾和恢复重建的重大胜利。地区生产总值达到1855亿元，同比增长23.6%；规模工业增加值620亿元，增长26.5%；固定资产投资1820亿元，增长22%；招商引资到位资金402亿元；社会消费品零售总额686亿元，增长18.5%；全口径财政收入192.3亿元，增长22.1%；地方级财政收入73.2亿元，增长20%；城市居民人均可支配收入17720元，农村居民人均纯收入6300元，分别增长14%和12%。

　　（一）积极转变发展方式，统筹推动“三化”进程，着力实施“三动”战略，全市经济呈现出又好又快的发展局面

　　大力实施投资拉动和项目带动战略，固定资产投资拉动作用明显加大。新建续建投资3000万元以上项目1607项，其中工业项目1151项，工业项目投资占全部投资的比重提高到66.9%。开工建设以千万吨炼油改造、百万辆整车生产基地、碳纤维产业基地、40万吨ABS等为代表的投资10亿元以上重大产业工程20项，以18万吨苯胺二期、吉福参科技人参深加工、吉林汽车冲压中心等为代表的投资亿元以上工业项目100项，其中千万吨炼油改造、300万吨精品钢一期、恒基多晶硅太阳能电池等7项重大产业工程和1万吨氯酰胺、10万套汽车天窗等45项重点项目竣工投产。快速推进现代服务业、城乡基础设施、社会民生事业等领域项目建设，欧亚商都、吉林站改扩建、人民医院一期等一批项目投入使用或基本具备运营条件。加强项目谋划，储备3000万元以上项目1000余项。

　　着力加速新型工业化，工业经济发展水平较大提高。化工、汽车、冶金等支柱优势产业产能扩大、层次提升，碳纤维及制品、先进装备制造等战略性新兴产业成长速度加快。完成规模工业总产值2130亿元。有效加快产业融合发展，化工、汽车产业配套协作发展规划编制完成，组织建设汽车、化工配套项目29项。哈达湾老工业区整体改造开始实施，冀东水泥搬迁已在永吉选址并开工建设。大力培育发展骨干企业和中小企业，一汽吉林汽车、康乃尔化工、明阳风电等重点企业主要装置负荷率大幅提高，百户重点工业企业产值增长25.2%；新增规模工业企业133户。下力气推进2009年建成项目达产达效，达产达效率达到80.8%。精心组织灾后生产恢复，219户受灾规模企业在较短时间内恢复生产。深入落实节能减排措施，超额完成省下达任务。创新驱动战略推进力度加大，国家级碳纤维高新技术产业化基地建设取得重大进展，省科技厅与我市工作会商机制启动运行，实施市级以上重大科技成果转化和科研攻关项目156项。

　　积极推进特色城镇化，城乡建设进程继续加快。突出城建十大工程等重大功能设施项目，人民广场、人民医院一期、女子学校重建一期、九中扩建、长春路改造及吉林站改扩建工程竣工使用或基本具备运营条件。松花江流域水污染治理工程基本完成，绿化、亮化和美化水平持续提升，新增绿地103公顷，硬覆盖15万平方米。西山国际花都等82个重点房地产项目开工建设，开发面积610万平方米，竣工335万平方米。“城市管理达标年”活动深入开展，市容市貌得到较大改善。磐石、桦甸、蛟河、舒兰城市扩容升级提速，永吉口前与市区对进式发展加快。岔路河、烟筒山、夹皮沟等强镇特色化发展优势更加突出。“千村示范、万村提升工程”快速实施，建成10个样板村、50个示范村和360个提升村。

　　加快推动农业现代化，县域经济实现新突破。现代农业产业体系加快建设。组织实施35个粮油高产万亩示范田创建工程，在严重受灾情况下粮食总产量仍达84亿斤；畜牧业和园艺特产业较快发展，新建市级以上标准化牧业小区47个，建设特色农产品示范区9个，畜牧业和园艺特产业产值均增长14.5%。农业产业化取得较大进展。着力建设中新（吉林）食品区，项目进区和启动实施步伐明显加快。建成投产3000万元以上产业化项目7个，市级以上龙头企业发展到156户，建成万昌和缸窑2个粮食加工转化示范乡镇。农田水利建设力度加大，建设各类水利设施2461项。农村改革不断深化。68个乡镇和涉农街道组建三资代理服务中心和便民服务中心，建立土地流转服务中心和服务站473个，新发展农民专业合作社280个。国有粮食购销企业产权制度和农村集体林权制度改革基本完成。五县（市）地区生产总值增长21.6%，地方级财政收入增长17.2%。

　　大力发展现代服务业，第三产业发展步伐进一步加大。商贸经济规模水平提高。中东二期大型综合商贸开发、世贸广场五星级酒店等10个重点商贸项目开工建设，奥亚名门购物广场等10个大型商贸设施投入运营；大力开展商贸企业晋档升级活动，新增207户企业进入限额以上行列。生产性服务业快速成长。内陆港口岸基础设施建设加快，东北亚、统泰等一批现代物流企业进一步发展。新成立3户担保公司和6户小额贷款公司，金融生态环境得到新的改善。旅游经济特色优势壮大。北大湖综合开发、圣德泉二期等重点旅游项目进展顺利，大型节庆活动水平提升，实现总收入163亿元，增长29.9%。

　　努力扩大对外开放，长吉经济一体化加快推进。精心组织参加世博会、东博会等大型经贸交流活动和赴鲁津、新加坡、台湾等境内外招商活动，一批知名企业落户我市，北京利华差别化醇醚、浙江新湖通田整车基地、深圳万科滨江新区等重大招商项目落地建设，全年新开工3000万元以上招商项目346项；实际利用外资4.5亿美元，增长22%。对外贸易持续发展，新增外贸进出口企业45户，实现进出口总额8.3亿美元。积极推动长吉一体化发展，两市战略合作框架协议正式签署，在产业融合、旅游合作等领域进行了富有实效的对接。吉林-深圳产业合作示范区开工重点项目8个，合作发展成效进一步显现。

　　（二）扎实办好民生事项，人民生活质量水平明显提高

　　把民生作为第一目标，从改善居住环境、出行条件和扩大就业等方面入手，进一步加大民生任务落实力度。新建创业市场4个，培训农村劳动力和下岗失业人员2.6万人，实现城镇新增就业9.5万人；棚户区、危旧楼拆迁73.5万平方米，6006户住房困难群众喜迁新居；建设廉租住房6.9万平方米，发放租赁补贴17883户；完成农村泥草房改造16387户；暖房子工程大力推进，取缔分散供热小锅炉63座，改造供热管网86.7公里，实施外墙保温310万平方米；住宅小区综合整治全面完成；新增城区天然气用户1.2万户，建设农村沼气池4235座，解决18.4万农村人口饮水安全问题；完成30所学校操场塑胶化建设；维修改造28条道路和16条街路照明设施；新建沙滩排球场2处、全民健身场地18处；新增居民休闲活动场所28万平方米；新农合筹资标准和报销比例提升。在此基础上，针对蔬菜价格变化，为城乡低保户、优抚对象、五保分散供养对象等适时发放价格补贴；针对城乡困难群体实际，将城市低保标准由每月人均240元提高到280元，农村低保标准由每年1320元提高到1420元；针对学生饮水问题，免费提供安全桶装饮用水；针对冬季雪量大、行路难，组织了清雪作业进小区。

　　（三）切实加大投入力度，各项社会事业实现全面进步

　　城乡优质均衡教育积极推进，基础教育全面加强。完成104所中小学校舍安全加固改造工程，农村薄弱校改造面积45.8万平方米；朝鲜族实验小学新校舍落成，民族教育水平得到新提高；职业教育成果显著，参加全国职业院校技能大赛获奖总数占全省61%；高等教育健康发展。城乡医疗卫生服务能力较大增强。农村三级医疗网络建设顺利完成；积极深化医药卫生体制改革，基层医疗机构基本药物使用试点扎实展开；加强传染病防控和卫生监管，公共卫生和医疗服务体系得到有效完善。积极

推进文化体制改革，文化体育事业蓬勃发展。市歌舞团连续参加央视春晚和世博会、亚运会等国家级大型演出；组织各类群众性文体活动100多项次，松花江之夏广场文化活动更趋繁荣，荣获国家群星奖。

修志工作填补6项历史文化空白，农家书屋和

广播电视村村通建设基本实现全覆盖。第六次全国人口普查顺利开展。新型农村社会养老保险试点工作稳步推进，社会保障体系建设水平进一步提高。

　　（四）有效强化安全稳定工作，平安吉林建设得到新发展

　　严格落实安全生产责任制，强化消防、生产、交通、食品药品等重点领域和行业安全隐患专项排查整治，切实增大安全投入，维护公共安全的长效监管体制和处置突发事件的应急救援体系进一步健全。认真完善信访工作责任制，集中解决了一批群众反映突出的难点问题。

强化社会治安综合治理，构筑起以公安机关为主体的街面动态防控网、以出租车司机为主体的快速机动防控网、以社会辅警力量和公益岗位人员为主体的社会面防控网，坚决打击各种犯罪活动，有力保障了社会的和谐稳定。不断加强人防工作，荣获全国人民防空先进城市称号。

　　（五）奋力抗击特大洪涝灾害，抗洪抢险救灾和恢复重建取得重大胜利

　　去年的特大洪涝灾害，袭击范围之广、造成破坏之强，史所罕见。我们在省委省政府的坚强领导下，党政军民齐心协力，干部群众团结一致，与这场自然灾害进行了艰苦卓绝的斗争。全力以赴抗洪抢险救灾。第一时间启动应急预案，集中力量迅速组织展开抢险救援行动。在很短时间内完成被困群众解救任务，因灾受伤人员得到及时治疗，受灾群众全部妥善安置，化工原料桶流入松花江等突发事件得到有效处置。千方百计保障灾区群众基本生活。积极开展灾区援助，真正做到了让受灾群众有饭吃、有水喝、有衣穿、有学上、有地方住、有病及时医治，做到了大灾之后无疫情，做到了供水、供电、通讯、交通等设施功能迅速恢复。快速扎实开展灾后恢复重建。立即出台20项政策措施，争分夺秒推进灾后恢复重建。6万多间水毁房屋得到重建或修缮，581处江河堤防等水利设施得到修复加固，228处水毁农村饮水工程恢复使用，432公里道路、555座便桥和17座桥梁恢复通行，受灾学校校舍和卫生院所按计划全部重建或维修，切实完成了“六个确保”的恢复重建任务。

　　各位代表，我们取得的这些成绩，是省委省政府和市委正确领导的结果，是市人大、市政协大力支持的结果，更是全市党政军民共同努力的结果。尤其在抗洪抢险救灾和恢复重建过程中，解放军指战员、武警消防官兵、民兵预备役人员、公安干警，各级干部和各族群众，众志成城、英勇奋战，涌现出许多可歌可泣的感人事迹。在此，我代表市人民政府，向辛勤工作在各条战线的广大建设者，向各级人大代表、政协委员、各民主党派、工商联和社会各界人士，向始终对吉林市发展给予支持帮助的中省直单位、兄弟城市和海内外朋友，表示诚挚的感谢！向为抗洪抢险救灾和恢复重建做出巨大贡献的广大干部群众、解放军指战员、武警消防官兵、民兵预备役人员和公安干警，以及医疗卫生工作者、新闻工作者、科技工作者和企业家，致以崇高的敬意！

　　总结一年来的工作，我们清醒地认识到，还存在不少矛盾和问题。一是加快转变经济发展方式，破解总量不足、结构不优的双重制约矛盾，打牢经济基础，实现更好更快发展面临的任务依然非常繁重。二是灾后恢复重建工作仍然十分艰巨，城乡综合防范和抵御自然灾害的能力亟待增强。三是安全生产还存在薄弱环节，必须持续加大整治和监管力度，切实创造一个良好的安全稳定环境。四是政府机关还需深入转变职能，不断提高办事效率和服务质量。对于这些矛盾和问题，我们要采取有效措施，认真予以解决。

　　二、“十二五”发展战略任务

　　“十二五”时期，是我市加快转变经济发展方式、全面建设小康社会的关键时期。尽管伴随全球经济结构深度调整还会出现许多不确定因素，但求和平、谋发展的时代主流和趋势没有改变，仍处于可以大有作为的重要战略机遇期。必须抓住一切有利条件，高举中国特色社会主义伟大旗帜，以邓小平理论和“三个代表”重要思想为指导，深入贯彻落实科学发展观，以科学发展为主题，以加快转变经济发展方式为主线，以经济结构战略性调整为重点，以科技进步和创新为支撑，以保障改善民生为根本出发点和落脚点，以改革开放为强大动力，统筹推进新型工业化、特色城镇化和农业现代化，大力实施投资拉动、项目带动、创新驱动战略和富民工程，坚持“四个坚定不移”，突出工业、全面发展，努力加快建设东北和东北亚重要的新型产业基地、旅游度假名城和生态宜居城市，使经济社会逐步走上以人为本、包容和谐、又好又快的科学发展道路。

　　主要预期目标：2015年，地区生产总值达到3750亿元；全口径财政收入占GDP比重达到17%；三次产业比重调整为6:51:43；城镇化率提高到63%；单位GDP能耗和二氧化碳排放均比2010年降低15%；城市居民人均可支配收入3.4万元，农村居民人均纯收入1.1万元。

　　——加快建设现代产业体系，率先实现经济发展方式转变的新跨越

　　把经济结构战略性调整作为主攻方向，加速推动经济转型升级，全面提高经济发展协调性和竞争力。一是全力加速新型工业强市建设。实施投资拉动、项目带动和创新驱动战略，做大做强支柱优势产业，改造提升传统产业，积极发展战略性新兴产业。遵循原料路线多元化、产品加工精深化、园区建设国际化的发展导向，快速推进化工循环经济示范园区建设，实现双千亿级产业发展目标；依托整车制造，以汽车零部件配套和汽车、化工产业融合发展为两翼，加快建设百万辆整车生产基地，跃上千亿级产业规模新台阶；坚持黑色、有色、炉料协同并举，着力实施产业升级改造，形成千亿级冶金产业基地；围绕发展高端农产品加工业，切实强化中新（吉林）食品区建设，快速打造具有国际竞争力的安全优质食品加工基地；立足区域资源优势，突出发展以水电、核电、风电为代表的清洁能源，建设千万千瓦级清洁能源示范基地；按照产业集群化发展要求，以提高资源综合利用水平为重点，快速壮大无机粉体环保纸、陶瓷、石材等非金属矿产加工业。加快发展以碳纤维及制品为核心的化工新材料产业，以核电风电装备、大型专用设备为核心的先进装备制造业，以电子元器件、工控电子、汽车电子为核心的基础电子和电力电子产业，以生物制药、生物化工为核心的生物技术产业，四大战略性新兴产业逐步成为支撑经济发展的先导产业。二是努力发展现代服务业。加快培育旅游支柱产业。围绕松花湖风景区保护性开发，高标准建设朱雀山城市森林公园、青山四季旅游度假基地，全面提升山水田园风光旅游；着力开发冰雪旅游，加快建设城区松花江雾凇观赏带、北大湖生态体验度假区等重点旅游项目；深度挖掘民俗、历史和特色文化旅游，重点完善龙潭山、小白山、陨石博物馆等景区景点，促进旅游资源优势向经济发展优势更快转化。积极发展文化产业。突出以电视剧制作为核心的传媒文化业、以歌舞团为基础的演艺娱乐业、以书法美术和民间工艺为主导的书画艺术业、以印刷出版为重点的印刷包装业发展，努力培育一批国内有影响的大型文化企业集团。加快发展现代物流业。规划建设北部工业物流基地、西南部汽车物流基地和西部农副产品物流基地，快速推进中新（吉林）食品区物流中心和县（市）级物流节点建设。改造提升商贸服务业。整合河南街、东市商贸区，联动发展城市核心商圈，推进城乡市场体系进一步完善。三是不断巩固农业基础地位。围绕农业现代化、农村城镇化、农民市民化，发挥金珠、孤店子、岔路河等示范乡镇的引领作用，有效推进现代农业示范工程建设。加强优质粮食基地建设，稳定提高粮食综合生产能力；着力发展现代牧业园区和牧业标准化小区，加快建立“一圈二带三大集中区”精品畜牧业发展格局；实施新一轮“菜篮子”工程，持续扩大园艺特产业，更加有效地构建现代农业产业体系。

　　——着力推动城镇化进程，加速实现城乡一体化协调发展的新跨越

　　把统筹城乡发展作为重要目标，加快城镇化推进速度，有效形成城镇带动农村、城乡共同繁荣的新格局。一是加速中心城市发展。坚持“北拓、南优、东控、西调”的方针，依托哈达湾老工业区整体改造，着力打造北部工业新区，促进城市工业重心合理北移；按照规划建设科学、城市功能完备、居住环境优美的目标，统筹建设南部新城，进一步形成“北工、中商、南居”的空间结构形态。启动轻轨工程规划，加快城市快速道路交通体系等重大基础设施建设，到2015年，基本具备承载250万人口的综合功能。二是积极推进长吉一体化框架下的城镇化发展。围绕“一环、三带、八大组团”的发展布局，重点建设长吉联结区域节点城镇，加快推动有条件的中心镇向小城市发展，壮大明城、江密峰、北大湖等一批工贸型、集贸型、旅游型特色城镇，加快构建以吉林市为核心，以县城和中心镇为节点的市域城镇群。三是发展提升区域次级中心城市。加强县级城市基础设施建设，全面提高承载能力和城市品位，5个县（市）综合经济实力在全省排名逐步前移，磐石、桦甸总体达到中等城市规模。

　　——努力发展社会民生事业，真正实现人民群众生活质量水平的新跨越

　　把改善民生作为根本目的，扎实推进富民工程，城乡居民过上更加殷实幸福的美好生活。一是着力完善基本公共服务体系。坚持优先发展教育，促进教育均衡发展，实现更高水平的普及教育和惠及全民的公平教育。不断增加文化、体育事业投入，重点建设人民大剧院、全民健身中心和传媒中心等公益性文化体育设施，切实提高历史文化名城的发展品位。加快城乡公共卫生体系和医疗服务体系建设，全面提升医疗保障水平和公共卫生服务能力。二是努力提高城乡居民收入水平。深入推动全民创业，大力促进充分就业，有效建立和谐劳动关系。坚持民富优先，合理调整收入分配关系，确保城镇居民收入增长幅度高于全国平均水平，农村居民人均纯收入保持全国前列。三是进一步健全社会保障和住房保障体系。城镇居民基本养老、医疗、失业、工伤、生育和新型农村养老保险2015年实现全覆盖。加大保障性住房工程和暖房子工程建设，城市人均住宅建筑面积达到30平方米，农村泥草房改造全部完成。

　　三、2011年工作安排

　　今年是实施“十二五”规划的第一年。我们要坚定不移地贯彻党的十七届五中全会和省委九届十一次全会、市委十一届十一次全会精神，牢固树立抢抓机遇和克难奋进意识，以推进科学发展为主题，以转变经济发展方式为主线，以保障改善民生为根本目的，坚持“三化”统筹，实施“三动”战略，确保全面实现“十二五”振兴发展的良好开局。

　　主要预期目标：地区生产总值达到2170亿元，增长17%；规模工业增加值740亿元，增长20%；固定资产投资增长25%；招商引资到位资金460亿元，增长15%；社会消费品零售总额828亿元，增长20%；全口径财政收入240亿元，增长25%；地方级财政收入88亿元，增长20%；城市居民人均可支配收入20200元，农村居民人均纯收入7060元，分别增长14%和12%。

　　（一）大力转变发展方式，在结构调整中推进经济又好又快发展

　　全力抓好项目建设。一是突出产业结构优化升级，滚动实施百万辆整车生产基地、碳纤维产业基地、丰满大坝重建等20个重大产业工程；推进6万吨环氧乙烷、核电管道装备、10万套汽车车桥总成、万吨钼酸铵等100个工业重点项目建设，建成投产超过40项。二是突出城乡综合承载功能有效增强，建设吉珲客运专线、吉林至沈阳和吉林至哈尔滨高速公路及北部工业新区基础设施、南部新城基础设施等一批城建交通重点项目。三是突出现代服务业提速发展，建设软银国际金融科技园、伯爵商贸综合开发、松花湖旅游度假基地等一批服务业重点项目。四是突出社会民生事业持续改善，建设人民医院二期、职业教育园区等一批社会民生重点项目。

　　着力推动工业经济快速增长。一是大力发展重点产业园区。坚持总体规划、分步开发，加快C4C5制丙烯、聚氨酯系列产品等一批重点项目建设，促进北部工业新区产业集群优势快速形成；围绕哈达湾老工业区整体改造，稳步、积极地推进水泥、造纸、炭素、铁合金等企业搬迁改造；依托现有产业基础，着力引入和建设大项目，加速提升化工循环经济示范园区总量规模和层次水平；下力量发展汽车产业园区，加快实施一汽30万辆整车扩能改造、新湖通田整车基地等重点项目，推动一汽森雅发动机装配、恒辉汽车改性料等一批整车配套项目开工建设。二是积极抓好化工、汽车产业融合发展。强化汽车专用料、车用化学品企业与一汽、吉化的有效衔接，不断扩大产品协作配套份额。三是培育发展骨干企业和中小企业。推进企业加大技改扩能，发展销售收入10亿元以上、5亿元以上、亿元以上企业分别达到18户、20户和60户；百户重点企业实现产值1400亿元，增长21%。实施新一轮民营经济腾飞计划，新增销售收入2000万元以上民营规模工业企业20户。四是快速推进建成项目达产达效。大力抓好千万吨炼油改造、300万吨精品钢一期、油页岩综合开发等去年建成项目达产达效，达产达效率提高到80%以上。五是强化生产指挥调度协调服务体系，设立工业发展基金，健全企业贡献评价机制，全面创新工业生产服务平台建设。六是扎实做好节能减排工作，确保完成省下达任务。

　　努力加大农业现代化步伐。一是加快发展现代农业。抓好10个万公顷优质玉米基地、6个万公顷优质水稻基地和4个千公顷优质大豆基地，粮食总产量稳定在90亿斤阶段水平。积极发展精品畜牧业，新建扩建200个标准化规模养殖小区，畜牧业产值增长10%。着力抓好桦甸野山参、蛟河黑木耳等5个特产业示范园区；实施“菜篮子”工程，建设3个市级棚膜蔬菜生产基地，园艺特产业产值增长11%。积极发展生态林业，建设农村公益林20万公顷以上。二是快速推进农业产业化。加快中新（吉林）食品区建设，开工种猪场、长白山特色产品等10个重点项目。建设宏粮米业大米深加工、新星公司花青素胶囊等20个农业产业化项目，年销售收入超亿元的农产品加工企业发展到25户。三是有效夯实农业发展基础。深入实施病险水库除险加固、中小河流综合治理、灌区配套改造等水利工程；加快建设市区和五县（市）农产品质量安全检测中心，农机化综合作业水平达到66%，村级信息服务站覆盖面扩展到75%。四是全面深化农村综合改革。积极落实强农惠农政策，建立乡镇农村土地流转服务中心38个，新增农民专业合作社200个，组建完成三资代理服务中心和便民服务中心20个，两个中心覆盖率达到100%。

　　加快发展现代服务业。一是进一步提高商贸经济发展水平。建设红星美凯龙家居城、成城汽贸电子城等10个重点项目，世贸商业广场、紫光苑大酒店等10个项目年内投入运营。继续抓好商贸企业晋档升级，培育销售额10亿元以上企业4户、5亿元以上10户、亿元以上16户。二是加快壮大旅游产业竞争优势。快速推动旅游重点项目建设，开发朱雀山、玄天岭公园，建设松花江水上旅游项目和松花湖卧龙潭等景区景观；深度开发旅游产品，多元开拓客源市场，实现旅游总收入206亿元。三是着力发展生产性服务业。加快完善现代物流网络体系，建设北部工业、西南部汽车产业和西部农副产品三大物流基地。强化投融资平台建设。引进2户股份制银行设立分支机构，加快农村商业银行筹建进程，推进永大集团挂牌上市。实施城市“一卡通”、数字社区等项目建设，促进信息服务业更快发展。

　　大力开展招商引资。一是强化主导产业招商。围绕支柱优势产业和新兴产业，下力气引进延伸产业链条、扩大配套加工的项目，力争新开工3000万元以上招商项目350项。二是发挥产业园区优势招商。依托北部工业新区、化工循环经济示范园区、汽车产业园区和中新（吉林）食品区等园区，着力在落地项目数量和质量上取得重大突破。三是突出重点企业招商。找准具有产业转移趋势的发达国家和地区，着力引进美国亨斯曼等一批国内外大企业；进一步深化与央企的交流合作，争取更多的央企项目落户我市。四是完善目标责任招商。健全招商引资机制，强化综合协调服务，切实提高项目履约率和资金到位率。

　　全面加强财税工作。一是强化财税收入征管服务。抓好涉税信息共享平台建设，推动国地税协作办税，进一步健全社会综合治税体系；加强对重点税源和新增税源的掌控分析，有效增强税收征管服务能力。二是努力提升财政收入质量。完善财政收入考核机制，科学优化收入结构，不断扩大地方级收入比重；加强行政事业单位存量资产等资源管理，有效盘活政府性资源。三是提高财政科学化精细化管理水平。深化财政改革，严格预算约束，合理扩大公共服务支出，把更多财力用于民生改善和经济社会发展上来。

　　（二）加快推进城镇化进程，大力构建长吉一体化框架下的城乡统筹发展格局

　　加强城市建设和管理，提升中心城市整体功能。规划实施城建重点项目145个。一是优化空间布局结构。结合哈达湾老工业区整体改造，统筹建设现代化新城区；整体开发城市南部、西南部，启动南部新城建设。二是继续抓好城建十大工程和基础设施建设。全面推进人民医院二期、吉林大街贯通等十大工程建设。加快建设金珠净水厂、吉钢大路、深东路等31个产业园区基础设施工程。新建改造3条污水管线，截流7个污水吐口，启动污水处理厂二期和再生水项目。抓好小白山、吉丰东线、东山等区域综合开发，房地产开工及续建600万平方米，竣工310万平方米。三是加强生态环境建设。实施城市绿化工程，恢复重建清水绿带，进一步提升一江两岸、主要景观路和广场的美化亮化水平。新增绿地70公顷。实施“城市管理创优年”活动。认真开展标准化社区建设试点工作，加速构建全社会参与城市管理的大城管格局，明显提升城市的环境质量和整体形象。

　　强化县城集聚功能，切实抓好小城镇和新农村建设。加强县城建设，增强综合承载功能和公共服务能力，逐步引导农村人口向县城转移。五县（市）城镇化率提高到41%。围绕长吉一体化发展，推动岔路河、桦皮厂、烟筒山、天岗等中心城镇加快向小城市发展，着力提升明城、江密峰、北大湖等重点特色镇的发展水平。深入实施“千村示范、万村提升”工程，进一步加快新农村建设步伐。

　　抓好灾后恢复重建，促进灾区加快发展振兴。以保春灌、保农民正常供水、保安全度汛为重点，扎实抓好损毁水库、灌区和温德河堤防等水利设施建设，力争基本恢复到灾前水平。加大施工组织力度，快速推进144座水毁桥梁、60公里道路的恢复重建。加快水毁耕地复垦进程，逐步恢复灾前耕种能力。加强应急抢险救灾物资储备和基础设施建设，不断提高防灾减灾救灾的整体能力。

　　（三）突出民生十项任务，扎实推进和谐社会建设

　　认真办好民生事项。（1）为人民群众提供良好创业就业环境。实施受灾群众就业援助计划，对有劳动能力和就业愿望的受灾群众，确保每户家庭至少一人安置就业。实施创业促就业推进工程，新发放小额担保贷款1亿元，扶持2000名创业者创业，带动就业15000人；对企业当年安置就业困难人员就业，签订劳动合同并缴纳社会保险费的，给予企业实际缴纳的基本养老、基本医疗和失业保险补贴；就业困难人员灵活就业、农民进城自主创业并以个人身份缴纳社会保险的，按实际缴纳社会保险费数额给予50%的社会保险补贴。实施城乡劳动者就业技能培训计划，按人均补贴880元标准，培训农村劳动力和城镇失业人员2.6万人；实施上岗技能无偿援助计划，免费培训退伍士兵、下岗职工、贫困人口和农村富余劳动力4000人以上。（2）改善人民群众居住条件。建设15万平方米廉租房和公租房，解决3000户住房困难群体居住问题；实施棚户区、危旧楼改造，拆迁建筑面积40万平方米以上，改善6000户居民住房；完成农村泥草房改造6485户。（3）实施暖房子工程。取缔分散供热小锅炉12座，改造热网80公里，继续推进墙体保温改造，加快供热网络联网步伐。（4）改进人民群众出行条件。建设雾凇西路、西山街等15条主次街路，维修改造滨江北路等8条主次街路和临江门大桥等4座桥梁。再更新100台公交车。（5）实施“菜篮子”工程。建设3个市级棚膜蔬菜生产基地，新建1200栋温室和大棚，新增蔬菜生产能力3500万斤。（6）实施农村“村村通”自来水工程，再解决15万农村人口安全饮水问题。（7）进一步解决群众就医难、看病贵问题。在市中心医院、人民医院、中西医结合医院实施基本药物零加价，市人民医院对本市（含五县市）居民实施大型医疗设施检查费下降20%。（8）实施养老援助计划。建设市级社会福利服务中心，改造和完善县（市）区级社会福利服务机构，进一步加强社区养老服务站建设。（9）丰富人民群众文化体育生活。北山公园免费向市民开放。打造松花江文化长廊，建设一批塑胶门球场、篮球场、羽毛球场和标准化全民健身场地；扶持10所学校装备校园健身设施和器械。开展公益免费电影放映活动，全年进社区、广场放映1000场，送电影下乡镇4000场，免费公演经典童话剧50场。举办首届松花江河灯文化节。（10）实施薄弱学校改造工程。对农村和城区75所中小学校舍实施重建或改造。

　　加快发展各项社会事业。继续实施创新驱动战略，加大力度建设国家级碳纤维高新技术产业化基地，推进大学科技园等创新平台建设，组织实施以突破碳纤维关键技术为核心的5项科技重大专项和10项重点科研攻关项目。深化教育资源整合，加强中小学校标准化建设，促进义务教育均衡发展；扶持30个职业教育重点专业，力争1—2所职业学校进入国家级示范校；推进学前教育，积极支持高校发展。深化医药卫生体制改革，整合医疗卫生资源，加快推进人民医院二期、中心医院扩建和妇婴医院建设；完善乡镇卫生院、村卫生室功能，有效促进乡村医疗卫生服务一体化管理。抓好以电视剧制作为核心的传媒文化业、以歌舞团为基础的演艺娱乐业及雾凇冰雪文化，进一步加快文化体育事业发展步伐。切实加强国防教育和国防动员，积极开展军地双拥双支活动，努力争创军民共建新成果。

　　扎实做好社会安全稳定工作。严格落实安全生产目标责任，强化长效监管机制和应急救援体系建设；加大安全生产和食品药品监管投入，全面提升综合防范能力，严防重特大事故，切实保障人民群众生命财产安全。积极做好信访工作，加强领导包案制度落实，妥善处理好人民群众的合理诉求。进一步完善社会治安防控体系，加快以城市报警和视频监控为龙头的“天网”工程和以居民区硬件防控设施为重点的“铜墙铁壁”工程建设；严厉打击各种严重刑事犯罪、经济犯罪和“法轮功”等邪教组织，更加有效地强化平安吉林建设。做好价格监管工作，努力保持消费品价格的基本稳定。

　　（四）加强政府自身建设，切实提高组织推进经济社会发展的实践能力

　　强化民主政治建设，进一步增强依法行政能力。自觉接受人大及其常委会的法律监督和政协的民主监督，做到重大工作事项报告、协商在前。广泛听取民主党派、工商联、无党派人士和各人民团体意见，不断加强和改进政府工作。进一步健全科学决策机制，凡事关经济社会发展全局的重大事项，都要坚持集体研究决定；完善民主决策程序，凡关系人民群众根本利益的重大决策，都要实行听证、论证和专家咨询制度；深化政府信息公开，凡不涉及国家秘密、商业秘密的政府信息，都要主动向社会公开，保证人民群众充分享有知情权、参与权、表达权和监督权。启动实施“六五普法”规划，全面贯彻国务院关于加强法治政府建设的意见，切实提高政府的依法行政水平。

　　强化政府职能转变，进一步提高公共服务效能。深化行政审批权相对集中改革，合理优化审批方式，再集中简化一批事项的审批环节；从清理收费事项入手，加大审批执法权规范和清理，再集中取消一批审批和收费事项，绝不允许乱收费蚕食发展成果，损害群众利益。健全“一站式”服务平台，扩大告知承诺和并联审批实施范围，有效提高办事效率，改善服务质量，努力营造少环节、高效率、低成本、零障碍的发展环境。进一步完善目标任务落实机制，各项重点工作全部纳入责任推进体系，切实做到职责明确，形成以实际成果评议机关及干部的激励机制和发展氛围。

　　强化勤政廉政建设，进一步树立政府良好形象。大力培塑开拓创新、求真务实的工作作风，引导政府公职人员特别是各级干部深入到基层和群众最需要的地方解决实际问题，使精力真正集中到狠抓落实上，使能力真正发挥到推动发展上。坚持廉洁办各项事业，充分发挥监察、审计等部门的监督职能，有效推进节约型机关建设。认真落实党风廉政建设责任制，扎实强化政府系统廉政风险防范管理，坚决依法查办违纪违法案件；深入完善政风行风测评制度，坚决纠正各种不正之风，努力建设为民、务实、廉洁、高效的服务型政府。

　　各位代表，“十二五”发展蓝图已经绘就，今年目标任务也已明确。我们一定在省委、省政府和市委的正确领导下，在市人大、市政协的大力支持下，更加紧密地团结起来，以奋发拼搏的高昂志气，开拓进取的旺盛精神，脚踏实地的务实作风，奋力再创全市经济社会发展的新辉煌！
